# Allergen‐specific subcutaneous immunotherapy for Japanese cedar pollinosis decreases the number of metachromatic cells and eosinophils in nasal swabs during the preseason and in season

**DOI:** 10.1002/iid3.301

**Published:** 2020-04-02

**Authors:** Kuninori Otsuka, Hirokuni Otsuka, Shoji Matsune, Kimihiro Okubo

**Affiliations:** ^1^ Otsuka ENT Clinic Yokohama Kanagawa Japan; ^2^ Otorhinolaryngology, Shin‐yurigaoka General Hospital Kawasaki Kanagawa Japan; ^3^ Otorhinolaryngology, Nippon Medical School Musashikosugi Hospital Kawasaki Kanagawa Japan; ^4^ Otorhinolaryngology and Head and Neck Surgery Nippon Medical School Tokyo Japan

**Keywords:** eosinophil, Japanese cedar pollinosis, mast cell, subcutaneous immunotherapy

## Abstract

**Background and objective:**

Nasal symptoms of allergic rhinitis can be reduced with allergen‐specific subcutaneous immunotherapy (SCIT). However, the mechanisms underlying the effectiveness of SCIT for Japanese cedar pollinosis are not well understood. We studied changes in the numbers of metachromatic cells, eosinophils and neutrophils in nasal swabs following SCIT for Japanese cedar pollinosis.

**Methods:**

Subjects were either untreated or given SCIT for 0.5 to 13 years duration. For the 2019 seasons, nasal swabs were taken in the pollinosis preseason (immunotherapy n = 36; untreated control, n = 62) and in the pollinosis season (immunotherapy n = 45; untreated control n = 46) and the numbers of mast cells, eosinophils and neutrophils assessed by microscopy.

**Results:**

There were significant improvements in symptom severities following SCIT in comparison to untreated subjects (*P* < .0003, the Mann‐Whitney U test) in preseason, and (*P* < .00001) in season. Metachromatic cell counts from nasal swabs of SCIT subjects in preseason and in the season were lower than those of untreated subjects (*P* = .0029 and *P* = .031, respectively). Eosinophil numbers in nasal swabs of subjects given SCIT were lower than in untreated subjects (*P* = .0031) in season, but not in preseason. There were no significant differences in degrees of neutrophilia between untreated and SCIT subjects in preseason and in season.

**Conclusion:**

One mechanism underlying the effectiveness of SCIT for Japanese cedar pollinosis involves a reduction in the number of metachromatic cells in nasal swabs in the preseason and an inhibition of increases in the number of metachromatic cells and eosinophils in season.

## Graphical abstract

The number of metachromatic cells in nasal swabs in untreated subjects (B) and SCIT (C) for Japanese cedar pollinosis in pre‐season and in season.

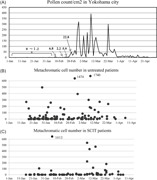

## 1 INTRODUCTION

Allergen‐specific immunotherapy for seasonal allergic rhinitis is a valuable treatment and an alternative to escalating the use of medications for symptoms. Moreover, it can have a long‐lasting effect, for example, up to 3 years following a single course of allergen‐specific immunotherapy.^1^

There are two methods of immunotherapy, subcutaneous (SCIT) and sublingual immunotherapy (SLIT). In previous double‐blinded, placebo‐controlled studies, SCIT was shown to be effective for reducing symptoms of seasonal allergic rhinitis to grass, ragweed, birch, and tree pollen.^2^, ^3^, ^4^

Japanese cedar pollinosis is the most common form of seasonal allergic rhinitis in Japan. In 2000, a standardized Japanese cedar extract for immunotherapy was introduced and determined to be effective.^5^ However, SCIT is not widely used in Japan because of the risk of anaphylaxis and side effects such as redness of the face or ears, itching or cough with wheezing.^6^, ^7^ Thus, there have been few reports of the use of SCIT in Japan. We have optimized our SCIT protocol to minimize side effects of the treatment and in the current study we compared the symptoms, and numbers of metachromatic cells (Mc, mast cells and basophils), eosinophils and neutrophils in SCIT subjects as compared with untreated subjects.

## 2 POLLEN COUNT

Japanese cedar pollen (JCP) counts were done every year in Yokohama using the Durham method.^8^ We defined the beginning of pollen dispersal as at least 2 consecutive days of 10 pollens/cm^2^ or over 20 pollens/cm^2^ on a single day.^9^ Pollen dispersal began on Feb 19th 2019 and was below 10 pollens/cm^2^ on 16th April (end of the season; Figure 2A). The cumulative JCP counts/cm^2^ in Yokohama was 3696 in 2019.

### 2.1 Subjects

All subjects were sensitized to JCP suffered from symptoms for over 3 to 30 years and were diagnosed as JCP seasonal allergic rhinitis according to Japanese Guidelines.^9^ During pollen season they had typical symptoms and eosinophilia in nasal swabs. They were positive for IgE antibodies to JCP with class 1 or more reactivity (Table 1). Subjects positive for IgE antibodies to house dust mite under class 1 without perennial symptoms, and subjects with positive IgE antibodies to orchard grass under class 4, but no symptoms in orchard season were included. They were negative for IgE antibodies to ragweed, mugwort, and *Alternaria*. All participants provided informed consent. The study was approved by the Ethics Committee of Nippon Medical School.

**Table 1:** Characteristics of subjects in the subcutaneous immunotherapy and untreated groups

SCIT		Untreated			
	In preseason+season	In preseason	*P* value	In season	*P* value
Number	33	62		45	
Age ± SE	54.8 ± 1.9	53.5 ± 1.5	.4	47.2 ± 2.3	.013
	34‐74	33‐81		25‐81	
M:F	10:23	17:45	.7	20:25	.2
SCIT duration, y	0.5‐13	…		…	
Average	5.6				
Class1,2,3,4,5,6 of JCP					
IgE: (n)	1,5,14,12,0,1	0,16,26,14,5,1	0.6	0,7,25,8,5,0	.9
Class1,2,3,4,5,6 of Orchard grass					
IgE (n)	1,1,3,1,0,0	0,1,0,1,0,0	0.01	3,4,0,1,0,0,	.8

*Note*: Each *P*‐value indicates comparison to SCIT subjects. There were significant differences on average age between SCIT and untreated subjects in season, and no differences in sexes for both preseason and in season groups.

Abbreviation: JCP, Japanese cedar pollens; SCIT, subcutaneous immunotherapy.

Thirty‐three Japanese cedar pollinosis subjects started to be treated with SCIT from 2006 to 2018 and continued during the preseason and/or in season without interruption, and 62 untreated subjects served as controls in preseason, and 45 different individuals as controls in season (Table 1). Thirty‐one of 33 SCIT subjects had their first clinic visit in preseason, and two had their first visit in season (Table 2). Five of 33 SCIT subjects had their second clinic visit in preseason and 27 in season. Nineteen had a third visit in season. In total there were 36 visits in preseason and 48 visits in the season for those who received SCIT. Of the untreated subjects, 62 visited the clinic in preseason and 45 visited in season for prophylactic and symptomatic treatment using pharmacotherapies. Until the SCIT subjects visited our clinic they did not receive any medication (confirmed through questionnaire). All SCIT subjects agreed not to take any medication, even if they had symptoms until the study was completed. They received medications following study completion if needed.

**Table 2:** Frequency and timing of clinic visits in the subcutaneous immunotherapy and untreated groups

	SCIT		Untreated	
	33		107	
Subjects (n)	January 15‐February 18 in preseason	February 19‐April 6 in season	January 15‐February 18 in preseason	February 19‐April 6 in season
Subject visits (n)	36	48	62	45
First visit	31	2	62	45
Second visit	5	27		
Third visit		19		

Abbreviation: SCIT, subcutaneous immunotherapy.

## 3 METHODS

### 3.1 Subcutaneous immunotherapy

Subjects commenced subcutaneous injections of 0.05 mL of 0.2 JAU/mL of standardized JCP extra (Torii, Co. Japan) and the volume injected was increased as outlined in Table 3 twice weekly until a 20 to 25 mm diameter swelling occurred at the skin injection site. Thereafter, they received the same amount of extract once a week for 4 weeks and once every 2 weeks for 8 weeks and then once a month all year long at least until study completion (Table 3). Using this protocol, the subjects had limited side effects and no anaphylaxis. Questionnaires comfirmed that SCIT was effective for symptoms in the season for the duration of SCIT.

**Table 3:** Schedule of subcutaneous immunotherapy

Intradermal injection was started and continued twice a week as below.						
0.2 JAU/mL	0.05, 0.07, 0.1, 0.15, 0.2, 0.3 mL					
2 JAU/mL	0.05, 0.07, 0.1, 0.15, 0.2, 0.3 mL					
20 JAU/mL	0.05, 0.07, 0.1, 0.15, 0.2, 0.3 mL					
200 JAU/mL	0.05, 0.07, 0.1, 0.15, 0.2, 0.3 mL					
Maintenance amount in 33 patients (keep the injection every month until this study is completed)						
Patients numbers	2	4	8	5	4	1
20 JAU, mL	0.05	0.07	0.1	0.15	0.2	0.3
Patients numbers	1	2	1	2	2	1
200 JAU, mL	0.05	0.07	0.1	0.15	0.2	0.3

*Note*: When the swelling at injected skin site was between 20 and 25 mm, the same amount of extra was used to inject once a week for four times, then once every two weeks for four times, and then continued once a month until this study.

Although moderate symptoms occurred in 2 of 36 (5%) SCIT subjects in preseason, they had no medication until the study was completed.

### 3.2 Safety of our SCIT protocol

The doses of allergen that we use in our protocol are necessary for the effectiveness of SCIT for JCP. However, a high dose carries with it a risk of side effects. Originally the protocol that we used for SCIT was based on the manual of SCIT used by the Nippon Medical School (see Table 3). However, when we commenced SCIT in our clinic in 1998, side effects following injections appeared two to three times a year. At that time, the maintenance dose for SCIT was determined when the local skin redness reaction was less than 50 mm after 15 minutes. Side effects included redness with the itch of face, arms, and legs or whole body, and wheezing. In all patients with such side effects, the swelling site (not redness) was more than 30 mm 15 minutes after the injection. Given these side effects, in 2006 we began to use a maintenance dose based on 20 to 25 mm of swelling at the skin injection site. We have had nobody with significant side effects or anaphylaxis using this latter approach.

### 3.3 Subjects' symptoms and severity grading

All subjects completed a detailed symptoms questionnaire each visit. Classification of symptom severity was done using the Japanese Guidelines.^9^ Frequency of sneezing attacks and of blowing the nose/day were categorized as: none, −; 1 to 5, +; 6 to 10, ++; 11 to 20, +++; and over 21, ++++. The severity of symptoms was a cumulative score for 1 week before the clinic visit.

Nasal obstruction was classified as: none −; feeling of obstruction but not requiring mouth breathing, +; strong obstruction requiring mouth breathing sometimes a day, ++; serious obstruction requiring mouth breathing most of a day, +++; and serious obstruction for the entire day, ++++. Severity grading was classified as: most severe; sneezing attacks or blowing the nose/day ++++ or nasal obstruction ++++, severe; sneezing attacks and blowing the nose/day +++ or nasal obstruction +++, moderate; sneezing attacks or blowing the nose/day ++ or nasal obstruction ++, mild; sneezing attacks or blowing the nose/day + or nasal obstruction+, none; no symptoms.

### 3.4 Analysis of cell types in nasal swabs

A single nasal swab was taken by a cotton applicator (tip 2 × 10 mm) and transferred to a glass slide for each subject on each visit. The slides were dried and the cells were fixed in 99% methanol for 3 minutes. They were stained using Hansel solution (Torii Co, Tokyo) for 1 minute.^10^ Neutrophils, eosinophils, and basophils were mainly observed in the mucous compartment of the swabs, whereas mast cells were observed among epithelial cells. The total numbers of neutrophils, eosinophils and Mc (mast cells and basophils) on each glass slide were assessed by a single trained observer (H.O. and K.O.) in a blinded fashion using a microscope and 200× magnification.^11^, ^12^, ^13^, ^14^ The numbers of neutrophils and eosinophils were graded as: −; none, ±: few scattered cells, 1+; found cells easily, 2+; abundant cells, with small clumps, 3+; abundant cells, often in clumps. The total number of Mc on the entire slide (Figure 2B, C) which was classified as: none, 1 to 9 cells, 10 to 99 cells, 100 to 999 cells, ≥1000 cells.

### 3.5 Statistical analyses

For comparison of groups, a *t* test for age, a *χ* ^2^ test for sex and the Mann Whitney U test for an anti‐IgE class, symptom severity, and cell infiltration were used. *P* < .05 was considered statistically significant.

## 4 RESULTS

### 4.1 Background of subjects

There was a significant difference in ages among the groups of SCIT and untreated subjects in season (*P* = .013), but not in preseason (*P* = .4) (Table 1). There were no significant differences in sex and classes of JCP IgE among SCIT and untreated subjects in preseason and in‐season. However, in classes of IgE to orchard grass, there was a significant difference among SCIT and untreated subjects in preseason (*P* = .01), although in all but one subject the amount of IgE to orchard grass was small (class 1).

### 4.2 Severity of symptoms between SCIT and untreated subjects in preseason and in season

**Figure 1:**
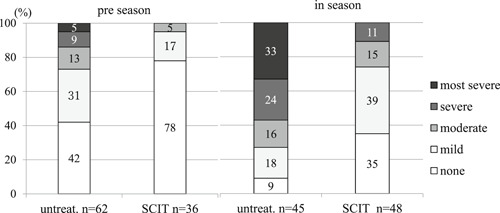
Symptom severity in untreated and SCIT subjects in preseason and season. There were significant differences in symptom severities between untreated and SCIT subjects (*P* = .0003, the Mann‐Whitney U test) in preseason, and (*P* = .00001) in season. SCIT, subcutaneous immunotherapy

The severity of symptoms among untreated and SCIT subjects was compared (Figure 1). In preseason, 95% of SCIT subjects had none or mild symptoms, whereas only 73% of untreated subjects had none to mild symptoms (*P* = .0003). In season 64% of SCIT subjects had none or mild symptoms and 26% of them had moderate to severe symptoms, whereas 27% of untreated subjects had none to mild symptoms and 74% of them had moderate to most severe symptoms (*P* = .00001).

### 4.3 Nasal cytology in SCIT and untreated subjects

**Figure 2:**
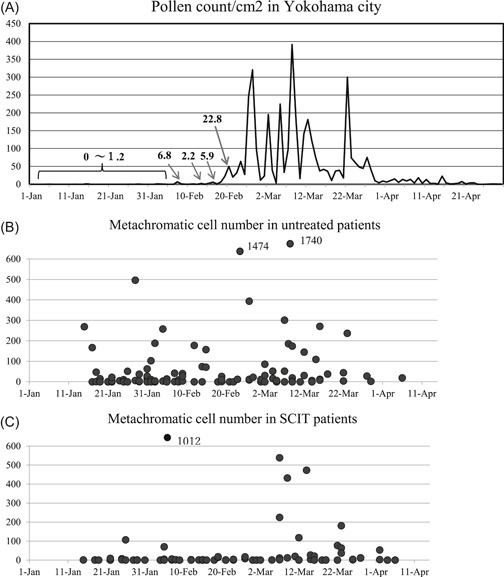
(A) Japanese cedar pollen daily counts (Durham method^8^) in Yokohama city, 2019. (B) Total metachromatic cell count in entire grass slide of untreated patients and (C) SCIT patients. SCIT, subcutaneous immunotherapy

**Figure 3:**
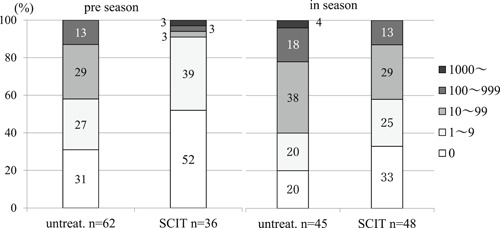
Proportions of untreated and SCIT subjects with different levels of metachromatic cells in nasal swabs in preseason and season. There were significant differences in metachromatic cells counts between untreated and SCIT subjects (*P* = .0029, the Mann‐Whitney U test) in preseason, and (*P* = .031) in season. SCIT, subcutaneous immunotherapy

Total metachromatic cell numbers in nasal swabs (Figures 2B, C and 3)

In preseason, proportions of untreated subjects with Mc numbers of 0, 1‐9, 10‐99, 100‐999, >1000 were 31%, 27%, 29%, 13%, and 0%, whereas those of SCIT subjects were 52%, 39, 3%, 3%, and 3%.

There were significant differences in metachromatic cells counts between untreated and SCIT subjects (*P* = .0029) in preseason.

In season the proportions of untreated subjects with total Mc numbers of 0, 1 to 9, 10 to 99, 100 to 999, >1000 were 20%, 20%, 38%, 18%, and 4%, whereas those of SCIT subjects were 33%, 25%, 29%, 13%, and 0%. There were significant differences in the number of Mc between untreated and SCIT subjects (*P* = 0.031) in season. The average ± standard error of Mc number in untreated subjects was 124 ± 49, whereas it was 50 ± 17 in SCIT patients, that is, 60% lower in SCIT subjects.

**Figure 4:**
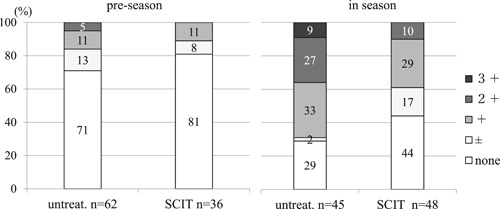
Proportion of untreated and SCIT subjects with various levels of eosinophilia in preseason and season. There was no significant differences in degrees of eosinophilia between untreated and SCIT subjects in preseason (*P* = .29). In contrast, there was a significant difference in degrees of eosinophilia between untreated and SCIT subjects in season (*P* = .0031, the Mann‐Whitney U test). SCIT, subcutaneous immunotherapy

Eosinophils in nasal swabs of untreated and SCIT subjects (Figure 4).

There were no significant differences in the degrees of eosinophilia between untreated and SCIT subjects in preseason. By contrast, in the season the proportion of untreated subjects with significantly more eosinophils in nasal swabs was greater than in SCIT subjects (*P* = .0031)

**Figure 5:**
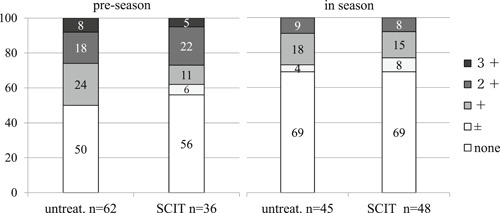
Proportion of untreated and SCIT subjects with various levels of neutrophilia in preseason and season. There were no significant differences in degrees of neutrophilia between untreated and SCIT subjects in preseason and in season. SCIT, subcutaneous immunotherapy

Neutrophils in nasal swabs of untreated and SCIT subjects (Figure 5).

There were no significant differences in degrees of neutrophilia between untreated and SCIT subjects in preseason and in season.

## 5 DISCUSSION

In 2008 over 30% of the Japanese suffered from JCP pollinosis.^9^, ^15^ The number of patients with JCP pollinosis is considered to be continually increasing. In 2018, the Welfare Department of Health published that 48.8% of citizens in Tokyo had JCP pollinosis.^16^

Before 2000, subcutaneous immunotherapy using a conventional extract with JCP allergen contained mainly protein Cry J2 (CryJ1 0.179 and CryJ2 2.39 μg/mL), and this extract had limited efficacy for immunotherapy.^17^, ^18^ Subsequently, an extract enriched for Cry J1 was reported to correlate with skin sensitivity better than that of Cry J2 and in 2000 the conventional extract was replaced by a standardized Cry J1 enriched extract (7.3‐21 μg/mL of Cry J1; 10 000JA U/mL, Torii Co, Japan) for SCIT.^19^, ^20^ Since this introduction of a standardized, Cry J1 enriched extract, there has been greater efficacy for SCIT than previously.^5^ Moreover, since in 2006 we began to use 25 mm as the upper limit for the local skin swelling reaction, rather than the previously recommended 50 mm skin red reaction, and we have seen no significant side effects of SCIT. However, in Japan the number of patients treated using SCIT has been few, because there has been a continuing fear of the occurrence of side effects such as anaphylaxis.^6^, ^7^ In JCP pollinosis, general treatment has been the administration of oral antihistamines, antileukotriene, and local steroid nasal spray.

In preseason and season of 2019 we compared the severity of symptoms, with the numbers of metachromatic cells, eosinophils and neutrophils in patients given SCIT and untreated patients. The severity of symptoms in SCIT subjects was significantly reduced in comparison to that of untreated subjects in preseason and in season. Moreover, for the first time, we show that there were significantly fewer Mc in SCIT subjects than in untreated subjects in both the preseason and in season.

There was no significant difference in degrees of eosinophilia between untreated and SCIT subjects in preseason (*P* = .29), but for the first time we show that there were significantly fewer eosinophils in nasal swabs of SCIT subjects in season. No differences were detected in the numbers of neutrophils in preseason or in season. The number of Mc and eosinophils in nasal scrapings were increased in season in all groups in comparison to offseason. However, between August and January, both cell types were still present in nasal scrapings.^21^

We reported the reduction of the nasal Mc number during HD/mite immunotherapy^22^ and others reported the reduction of eosinophil cationic protein and tryptase in nasal secretions following grass and ragweed SCIT.^23^ On the other hand, Wilson et al,^24^ reported that grass pollen immunotherapy for 2 years inhibits seasonal increases in basophils and eosinophils in the nasal epithelium, but no changes in mast cell numbers. Nouri‐Aria et al^25^ studied the effects of 2 years of SCIT in grass pollen allergic rhinitis and showed that SCIT reduced numbers of mast cells and the mast cell growth factor IL9 protein and mRNA in the nasal mucosa. We previously studied smears of dispersed cells from nasal epithelial surface of 10 patients with allergic rhinitis and found that the mean of the proportion of tryptase only, tryptase/chymase positive, and protease negative (possibly basophils) metachromatic cells were 82.8%, 10.1%, and 7.1%, respectively.^26^ In the current study, the number of metachromatic cells decreased by 60% in SCIT patients compared with untreated patients, suggesting that this decrease in metachromatic cell number was mainly in mast cells.

Previously, Otsuka et al^27^ and Kim et al^28^ reported that nasal epithelial cells express mRNA and protein for the mast cell growth factor, SCF and that SCF expression correlated with mast cell numbers in nasal scrapings. SCF stimulation of mast cell proliferation can be enhanced by other cytokines including IL‐4.^29^, ^30^ Lorentz and Bishoff reported on the effects of SCF and IL4 on the survival and phenotypes of mature human mast cells isolated from intestinal tissues. IL4 strongly enhanced tryptase +ve mast cell numbers (the most predominant mast cell in nasal scrapings), whereas SCF alone supported the predominance of tryptase/chymase +ve mast cells.^30^

Several previous studies using SCIT with JCP reported reductions in IgE levels and basophil activation, increases in IgG4, Treg, and Breg, and suppression of IL‐5 and IL‐4‐producing Th2 cell number.^5^, ^31^, ^32^, ^33^ In studies of the effects of SCIT using other pollens, there are reports of enhanced IgG and IgG4 antibody responses,^34^, ^35^ shift from Th2 to Th1 responses, reduced T cell produced IL4, IL13, and IL5,^23^, ^36^ inhibition of allergen‐IgE binding to B cells,^37^, ^38^ reduced basophil activation,^39^, ^40^ and reduced basophil, eosinophil numbers in the nasal mucosa, and eosinophil cationic protein and tryptase in nasal secretions.^41^, ^42^ The current study is the first report of the reduction in numbers of metachromatic cells and eosinophils in nasal swabs following SCIT. The mechanisms underlying these reductions in cell numbers require further investigation, perhaps with a focus on levels of IL4, IL9, and IL5.

In summary, we conclude that there is a reduction in the numbers of metachromatic cells and eosinophils in nasal swabs of SCIT subjects given a safe but effective dose of JCP compared with untreated subjects.

## CONFLICT OF INTERESTS

The authors declare that there are no conflict of interests.

## Data Availability

The authors confirm that the date supporting the findings of this study are available within the article and on request from the corresponding author.
